# Cold Sensitivity in Ulnar Neuropathy at the Elbow - Relation to Symptoms and Disability, Influence of Diabetes and Impact on Surgical Outcome

**DOI:** 10.3389/fcdhc.2021.719104

**Published:** 2021-08-16

**Authors:** Malin Zimmerman, Hanna Peyron, Ann-Marie Svensson, Katarina Eeg-Olofsson, Erika Nyman, Lars B. Dahlin

**Affiliations:** ^1^ Department of Orthopaedics, Helsingborg Hospital, Helsingborg, Sweden; ^2^ Department of Translational Medicine – Hand Surgery, Lund University, Malmö, Sweden; ^3^ National Diabetes Register, Centre of Registers, Gothenburg, Sweden; ^4^ Department of Molecular and Clinical Medicine, Institute of Medicine, University of Gothenburg, Gothenburg, Sweden; ^5^ Department of Medicine, Sahlgrenska University Hospital, University of Gothenburg, Gothenburg, Sweden; ^6^ Department of Biomedical and Clinical Sciences, Linköping University, Linköping, Sweden; ^7^ Department of Hand Surgery, Plastic Surgery and Burns, Linköping University Hospital, Linköping, Sweden; ^8^ Department of Hand Surgery, Skåne University Hospital, Malmö, Sweden

**Keywords:** ulnar nerve, ulnar nerve compression neuropathy, patient report outcome measure (PRO), diabetes, diabetic neuropathy

## Abstract

Cold sensitivity, an abnormal response to exposure to cold, is debilitating. It often affects people with nerve injuries and diabetes. Knowledge about the occurrence and prognostic impact of cold sensitivity in people with ulnar neuropathy at the elbow (UNE) is limited. We aimed to investigate the occurrence of cold sensitivity in UNE in relation to disability, the influence of diabetes and impact on surgical outcome. Data concerning 1270 persons operated on for UNE from 2010-2016 from the Swedish National Register for Hand Surgery (HAKIR) were matched with data from the Swedish National Diabetes Register (NDR). Disability and symptoms were assessed preoperatively, and at three and 12 months postoperatively using QuickDASH and a symptom-specific survey (HQ-8) containing one item regarding cold sensitivity. Differences regarding grade of cold sensitivity, occurrence of diabetes, QuickDASH scores and HQ-8 scores were studied. A linear regression analysis was performed to predict surgical outcome based on preoperative cold sensitivity. The mean age of the cases was 52 ± SD 14 years and 48% were women. Preoperatively, 427 answered the questionnaire. Severe cold sensitivity was present in 140/427 (33%) cases, moderate in 164/427 (38%) and mild in 123/427 (29%) cases. Cases with severe preoperative cold sensitivity reported higher QuickDASH scores at all times compared to cases with mild cold sensitivity. Relative change in QuickDASH scores over time did not differ between the groups. Cases with diabetes reported worse cold sensitivity preoperatively, but not postoperatively. All HQ-8 items improved with surgery, but cases with severe cold sensitivity reported worse persisting symptoms. Cold sensitivity is a major problem among those with UNE and an even greater preoperative problem among people with diabetes. It is associated with more symptoms and disability pre- and post-operatively. All cases, regardless of preoperative degree of cold sensitivity improve with surgery.

## Introduction

Cold sensitivity, an abnormal painful response to cold exposure, is a debilitating condition, which often occurs after hand trauma, as a result of peripheral nerve injuries, and in carpal tunnel syndrome (CTS) (1). Cold sensitivity is also a symptom of ulnar neuropathy at the elbow (UNE) (2). UNE has an incidence rate of 21-30/100 000 person-years (3, 4). It occurs when the ulnar nerve becomes compressed at elbow level for example beneath the Osborne’s ligament. When the nerve is compressed disruptions occur in the intraneural microcirculation, leading to epineural oedema and dynamic ischemia, inhibition of axonal transport and subsequent structural changes in the nerve fibres with axonal degeneration. The ischemia triggers early symptoms of UNE, such as paraesthesia and numbness, and later permanent sensory and motor loss may be seen, possibly caused by axonal degeneration (5). The structural axonal alterations in UNE, which may involve nerve fibres of different sizes, i.e. myelinated and non-myelinated nerve fibres, and their relation to the pathophysiology of cold sensitivity, remain unknown (6). Cold sensitivity is more common in people with diabetes, BMI >25, rheumatic diseases and in women (2, 7). It is associated with reduced quality of life and characterized by pain, stiffness, weakness, numbness and colour changes in the affected body part (8, 9). The causes for the occurrence of cold sensitivity in UNE and the influence of diabetes as well as the effects of surgery are not entirely clear. The aim of this study was to investigate the presence of cold sensitivity in those with UNE at the elbow requiring surgical treatment, its relation to other symptoms and disability, the influence of gender and diabetes, as well as the effects of surgical treatment.

## Methods

### Data Sources

All patients ≥18 years operated on for UNE (identified through the ICD-10 code G562 and surgical KKÅ97 codes ACC53, ACC43 or NCK19) from 2010-2016 registered in the Swedish National Quality Register for Hand Surgery (HAKIR) were included in the study. Patients completed the Swedish version of the QuickDASH questionnaire (10) and a symptom questionnaire (HAKIR questionnaire-8, HQ-8) before and three and 12 months after surgery (11). If the questionnaires were not digitally returned after two days, one reminder was sent by text message (12). The HQ-8 questionnaire comprises eight questions concerning: pain on load; pain on motion without load; pain at rest; stiffness; weakness; numbness; cold sensitivity; and ability to perform daily activities (11). The answers to each question are reported on a Likert scale. Check boxes are marked with 0, 10, 20…100; i.e. 100 signifies the worst possible perceived symptom. Based on answers to the relevant question in HQ-8, we have earlier defined three degrees of severity of cold sensitivity; mild (score <30), moderate (30–70) and severe (>70) (1). The QuickDASH is an 11-item questionnaire which provides overall information about the patient’s perceived disability in upper extremity musculoskeletal disorders. It is a shortened version of the well-established 30-item DASH-questionnaire and is considered similarly accurate (13). Each question is scored one to five. A total score for the entire questionnaire ranges from zero to 100, where 100 signifies the worst possible disability. The presence of diabetes at surgery and preoperative data on HbA1c levels were collected from the NDR. The NDR was started in 1996 and today includes the majority of people with diabetes in Sweden (14). Personal identification numbers were used to link data from the HAKIR and the NDR.

### Statistics

Normally distributed data are presented as mean ± standard deviation (SD); non-parametric data as a median [interquartile range, IQR] and nominal data as a number (%). For comparisons of nominal data, we used the Chi-squared test. The Kruskal Wallis test and Mann-Whitney U test were used for group comparisons. Linear regression analysis was used to study the effect of cold sensitivity on QuickDASH scores and the effect of diabetes on cold sensitivity scores. The linear regression analyses were adjusted for age, sex and diabetes (when applicable). Spearman’s correlation was used to assess correlation between preoperative cold sensitivity and preoperative total QuickDASH score. We interpreted an r-value of 0.30-0.70 as a moderate correlation and >0.70 as a strong correlation. Each operated arm was considered a separate statistical entity. A p-value of <0.05 was considered statistically significant. IBM SPSS Statistics (version 27; SPSS Inc., Chicago, IL, USA) was used for all statistical calculations and spider diagrams were created in Microsoft Excel for Mac, version 16.16.27 (Microsoft).

### Ethics

The Regional Ethics Review Board in Lund, Sweden approved the study (2016/931, 2018/57 and 2018/72). Everyone provides informed consent before being registered in HAKIR and NDR.

## Results

### Study Population

In total, 1354 surgeries for UNE were identified in HAKIR during the given period. Eight cases were excluded as they were under 18 years of age. As 76 persons were operated on bilaterally, our study included 1346 cases, from 1270 people, where the ulnar nerve was subjected to surgery at the elbow. Mean age at surgery was 52 ± SD 14 years and 649/1346 (48%) were women (**Table 1**). Concomitant procedures were performed in 201/1245 (16%) cases (data were missing in one case). The most common concomitant procedures were carpal tunnel release (85/201, 42%), trigger finger release (15/201, 7%) and surgery for ulnar nerve compression in Guyon’s canal (14/201, 7%). More than half of the cases, 735/1346 (55%), underwent surgery during the winter period (October-Mars), and 611/1346 (45%) during the summer period (April-September).

**Table 1:** Characteristics and cold sensitivity scores in persons with diabetes and ulnar neuropathy at the elbow (UNE) compared to persons without diabetes and UNE.

	All (n = 1346)	No diabetes (n = 1186)	Diabetes (n = 160)	P-value
Age, years	52 ± 14	51 ± 14	60 ± 11	**<0.0001**
Women, n (%)	649 (48)	586 (49)	63 (39)	**0.018**
Concomitant procedures, n (%)	116 (9)	92 (8)	24 (15)	0.225
Mild cold sensitivity (<30) preoperative n (%)	123/427 (29)	115/384 (30)	8/43 (19)	0.16
Moderate cold sensitivity (30–70) preoperative n (%)	164/427 (38)	153/384 (40)	11/43 (26)	0.071
Severe cold sensitivity (>70) preoperative n (%)	140/427 (33)	116/384 (30)	24/43 (56)	**0.001**
Preoperative cold sensitivity score	60 [20-80] (n=427)	55 [20-80] (n=384)	76 [39-91] (n=43)	**0.006**
3 months postoperative cold sensitivity score	20 [1-60] (n=319)	20 [1-60] (n=282)	20 [4-54] (n=37)	0.69
12 months postoperative cold sensitivity score	30 [3-70] (n=305)	30 [3-70] (n=265)	40 [1-70] (n=40)	0.82
Change in cold sensitivity score 0-12 months	3 [-5-31] (n=113)	2 [-5-31] (n=101)	16 [-14-52] (n=12)	0.63

P-value for comparison between cases with diabetes and cases without diabetes. Bold values illustrate statistical significance (p<0.05).

### Responders *vs.* Non-Responders

Response rates preoperatively and at three and 12 months postoperatively were 451/1346 (34%), 325/1277 (25%) and 307/1081 (28%), respectively. There were no differences in diabetes prevalence between responders and non-responders at any time point (data not shown). At three months postoperatively, responders were slightly older than non-responders. At 12 months postoperatively, there were more women among the responders than among non-responders and responders were a little older than non-responders (data not shown).

### Persons With and Without Diabetes

The 1346 cases included in the cohort comprised 160/1346 (12%) persons with diabetes at the time of surgery (**Table 1**). Those with diabetes reported greater preoperative cold sensitivity scores (76 [39-91]) than those without diabetes (55 [20-80]; p=0.006), but there were no postoperative differences (**Table 1**). In the linear regression analysis, diabetes predicted a higher preoperative cold sensitivity score [unstandardized B 12.5 (1.7-23.7), p=0.023)], but diabetes had no effect on postoperative scores (three months postoperative unstandardized B -0.71 (-12.1-10.7); p=0.90 and 12 months postop -2.15 (-13.5-9.2); p=0.71). We found no differences in HbA1c values, retinopathy frequency, BMI or duration of diabetes between persons with diabetes and moderate or severe cold sensitivity compared to those with diabetes and mild cold sensitivity (data not shown).

### Severity of Cold Sensitivity

Compared to cases with mild cold sensitivity, cases with severe cold sensitivity scored higher on the QuickDASH questionnaire at three and 12 months postoperatively (**Table 2**). Change in QuickDASH scores from preoperative to postoperative did not differ between groups (**Table 2**). In the linear regression analysis, moderate and severe preoperative cold sensitivity predicted higher preoperative QuickDASH scores compared to mild cold sensitivity [unstandardized B 11.8 (95% CI 7.2-16.4); p<0.0001 and 23.3 (18.3-28.2); p<0.0001]. Severe preoperative cold sensitivity predicted higher QuickDASH scores at three [unstandardized B 21.6 (10.7-32.5); p<0.0001] and 12 months postoperatively [unstandardized B 18.3 (6.9-29.8); p=0.002] than mild preoperative cold sensitivity. Moderate, compared to mild, preoperative cold sensitivity did not predict higher QuickDASH scores at three months [unstandardized B 6.5 (-3.7-16.7; p=0.21], but did at 12 months postoperatively [unstandardized B 11.3 (0.24-22.3; p=0.045]. All regressions were adjusted for sex, age at surgery and diabetes at surgery. Cold sensitivity scores did not differ between persons with concomitant nerve surgery and the rest of the study population at any time point.

**Table 2:** Characteristics and QuickDASH (short version of the Disabilities of the Arm, Shoulder and Hand) scores of cases with mild, moderate, and severe cold sensitivity treated for ulnar neuropathy at the elbow (UNE).

Characteristics and QuickDASH	Mild cold sensitivity (n = 139)	Moderate cold sensitivity (n = 148)	Severe cold sensitivity (n = 140)	P-value
Age, years	52 [43–61]	51 [41–61]	55 [44–64]	0.06
Women n (%)	55 (40)	66 (45) ns	81 (58)^B^	**0.007**
Diabetes n (%)	9 (6)	10 (7) ns	24 (17)^B^	**0.003**
QuickDASH preoperatively	32 [18–52] (n=139)	50 [36–64]^A^ (n=147)	63 [50–75]^B^ (n=136)	**<0.0001**
QuickDASH 3 months postoperative	11 [2–33] (n=42)	25 [9–44]^A^ (n=46)	45 [19–69]^B^ (n=42)	**<0.0001**
QuickDASH 12 months postoperative	15 [7–46] (n=34)	34 [17–53]^A^ (n=37)	45 [23–70] ns (n=39)	**0.003**
Change in QuickDASH 0–12 months	8 [-3–20] (n=34)	7 [-1– 21] (n=37)	14 [0–27] (n=39)	0.373

Groups were based on preoperatively scored cold sensitivity in HAKIR questionnaire-8 (HQ-8); mild (cold sensitivity score ≤ 30), moderate (31–70) and severe (>70). Data are presented as number (%) and median [interquartile range (IQR)]. Bold values illustrate statistical significance (p < 0.05) somewhere between the three groups, assessed with Kruskal Wallis for continuous variables and with Chi-square for nominal variables. The prefixes ^A^ and ^B^ indicate statistical significance (p < 0.05) between mild and moderate, and moderate and severe, respectively. ns means non-significant. The Mann-Whitney test was used for significance testing for continuous variables and Chi-square for nominal variables when comparing the groups two and two.

### Cold Sensitivity, QuickDASH and HQ-8

**Figure 1:**
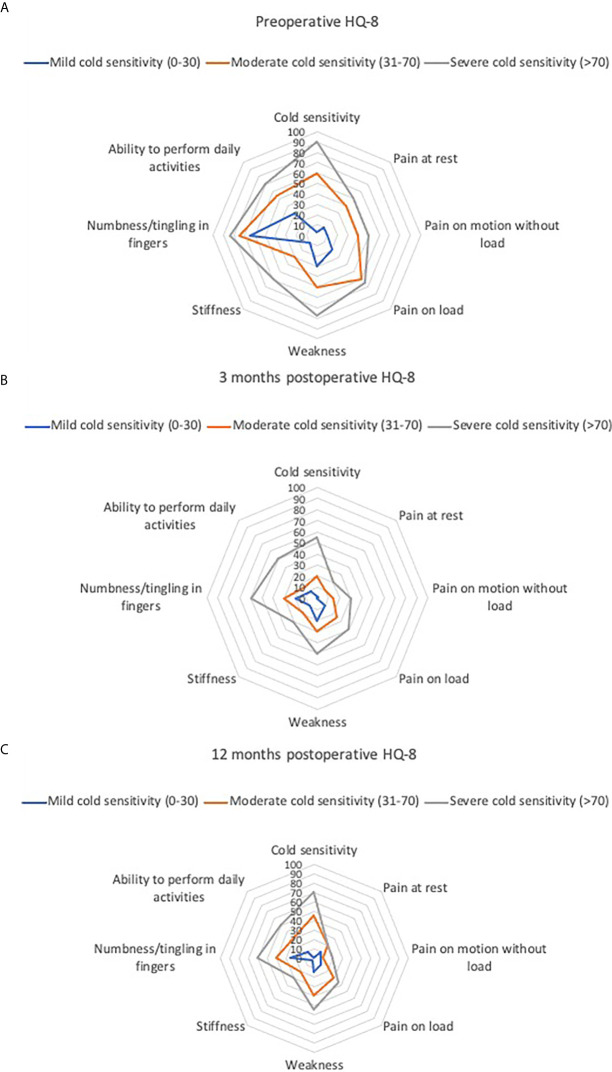
**(A–C)** HAKIR Questionnaire (HQ-8) scores in persons treated for ulnar neuropathy at the elbow (UNE) with mild, moderate, and severe cold sensitivity. Groups based on preoperatively scored cold sensitivity in HAKIR questionnaire (HQ-8), mild (cold sensitivity score ≤ 30), moderate (31–70) and severe (>70). **(A)** preoperatively, **(B)** 3 months postoperatively and **(C)** 12 months postoperatively.

We found a moderate correlation between preoperative cold sensitivity and preoperative total QuickDASH score (r = 0.47; p<0.0001). Preoperatively, persons with moderate and severe cold sensitivity scored higher on all HQ-8 items than those with mild cold sensitivity (**Table 3** and **Figure 1A**). Three and 12 months postoperatively, persons with moderate and severe cold sensitivity still scored higher on most of the HQ-8 items compared to those with mild cold sensitivity (**Table 3** and **Figures 1B, C**). At 12 months postoperatively, the cold sensitivity item was the only HQ-8 item with significantly higher scores in the severe group compared to the moderate group (**Table 3**).

**Table 3:** Patient reported scores on HAKIR questionnaire (HQ-8), preoperatively, three and 12 months postoperatively, in cases with mild, moderate, and severe cold sensitivity treated for ulnar neuropathy at the elbow (UNE).

HQ-8 items	Preoperative			P-value	3 months postoperative			P-value	12 months postoperative			P-value
	Mild cold sensitivity	Moderate cold sensitivity	Severe cold sensitivity		Mild cold sensitivity	Moderate cold sensitivity	Severe cold sensitivity		Mild cold sensitivity	Moderate cold sensitivity	Severe cold sensitivity	
**Cold sensitivity**	3 [0-20] (n=139)	60 [47-70] (n=148)^A^	90 [80-96] (n=140)^B^	**<0.0001**	2 [0-10] (n=40)	20 [10-50] (n=44)^A^	55 [13-80] (n=40)^B^	**<0.0001**	0 [0-20] (n=35)	45 [9-73] (n=38)^A^	70 [40-80] (n=40)^B^	**<0.0001**
**Pain at rest**	10 [0-41] (n=138)	40 [17-60] (n=147)^A^	50 [27-70] (n=137)^B^	**<0.0001**	1 [0-13] (n=42)	10 [0-30] (n=46)^A^	20 [0-60] ns (n=42)	**0.004**	10 [0-30] (n=36)	20 [10-50] (n=38)	20 [1-50] (n=40)	0.051
**Pain on motion without load**	10 [0-32] (n=138)	40 [12-57] (n=146)^A^	50 [22-70] (n=137)^B^	**<0.0001**	0 [0-16] (n=42)	14 [0-30] (n=46)^A^	30 [1-61] ns (n=41)	**<0.0001**	7 [0-25] (n=36)	10 [10-49] (n=37)^A^	20 [5-40] ns (n=39)	**0.032**
**Pain on load**	20 [2-60] (n=138)	60 [30-71] (n=146)^A^	65 [43-80] (n=137)^B^	**<0.0001**	10 [0-30] (n=41)	25 [10-49] (n=46)^A^	40 [10-70] ns (n=41)	**<0.0001**	10 [0-50] (n=36)	30 [10-68] (n=37)	36 [20-68] (n=40)	0.055
**Weakness**	30 [10-60] (n=138)	50 [30-78] (n=146)^A^	77 [59-90] (n=138)^B^	**<0.0001**	20 [0-34] (n=42)	30 [10-60] (n=45)^A^	50 [20-75] (n=41)^B^	**<0.0001**	15 [1-40] (n=36)	40 [18-64] (n=38)^A^	54 [30-70] ns (n=40)	**0.002**
**Stiffness**	10 [0-33] (n=136)	30 [5-50] (n=148)^A^	60 [33-80] (n=137)^B^	**<0.0001**	10 [0-22] (n=42)	18 [0-30] (n=46)^A^	30 [3-61] ns (n=42)	**0.003**	2 [0-30] (n=36)	20 [0-40] ns (n=38)	30 [3-50] ns (n=39)	**0.022**
**Numbness/tingling in fingers**	65 [37-80] (n=139)	75 [60-90] (n=148)^A^	84 [75-92] (n=139)^B^	**<0.0001**	20 [7-50] (n=42)	30 [10-60] ns (n=46)	60 [20-87] (n=42)^B^	**0.001**	25 [10-55] (n=36)	40 [18-80] (n=38)^A^	60 [30-89] ns (n=41)	**0.003**
**Ability to perform daily activities**	30 [4-60] (n=139)	54 [33-71] (n=147)^A^	70 [50-86] (n=140)^B^	**<0.0001**	8 [0-33] (n=42)	15 [0-53] ns (n=46)	50 [10-73] (n=42)^B^	**<0.0001**	10 [0-48] (n=36)	30 [10-60] ns (n=38)	50 [10-60] ns (n=41)	**0.021**

Groups were based on preoperatively scored cold sensitivity in HQ-8, mild (cold sensitivity score ≤ 30), moderate (31–70) and severe (>70). Data presented as (numbers, n) and median [interquartile range (IQR)]. Bold values illustrate statistical significance (p<0.05) somewhere between the three groups, assessed using Kruskal Wallis. The prefixes ^A^ and ^B^ indicate statistical significance (p<0.05) between mild and moderate, and moderate and severe, respectively; ns means non-significant. The Mann-Whitney test was used for significance testing when comparing the groups two and two.

## Discussion

Cold sensitivity is a common symptom in people requiring surgical treatment for UNE and an even greater preoperative problem among those with diabetes. Since we saw an association and correlation between preoperative cold sensitivity and high preoperative QuickDASH score, our proposal is that cold sensitivity is strongly related to impaired hand function. Overall, this corresponds well to other studies investigating the impact of cold sensitivity (6, 8). People with moderate or severe cold sensitivity before surgery had worse symptoms and more disabilities after surgery than those who reported mild cold sensitivity preoperatively. However, the change in QuickDASH scores from preoperative to postoperative did not differ between groups, indicating that the absolute improvement after surgical treatment is similar. Hence, there is no reason to avoid surgery in persons with severe cold sensitivity, but they should be informed about the risk of enduring symptoms. People with diabetes did not exhibit more extensive symptoms of cold sensitivity after surgery, in contrast to surgically-treated carpal tunnel syndrome, where those with type 1 and 2 diabetes have more cold sensitivity symptoms at one year, but not at five, postoperatively (15, 16), indicating that people with diabetes have a delayed recovery.

To achieve the best possible symptom relief after surgery, early surgery in persons with UNE and moderate or severe cold sensitivity might be preferable, in order to hinder the progression of cold sensitivity and restore hand function as soon as possible. Further, we want to emphasize that persons with UNE benefit from surgery irrespective of the grade of cold sensitivity and, therefore, surgery should be considered even in cases with a severe level of cold sensitivity.

Cold sensitivity in nerve compression syndromes seems more common than was previously thought. If our study is assumed to represent all persons with UNE, 67% suffer from moderate or severe cold sensitivity, which is a substantial number. In a previous study, we investigated cold sensitivity in carpal tunnel syndrome (CTS), also by using QuickDASH and HQ-8 questionnaires. The corresponding percentage of people with CTS with moderate and severe cold sensitivity preoperatively was 60% (1). This indicates a similarly high occurrence of cold sensitivity in the two most common nerve compression syndromes in the upper extremity. Another small study (n=100), using the Cold Intolerance Symptom Severity (CISS) questionnaire to evaluate the occurrence of cold sensitivity in both UNE and CTS, found that 52% of those included suffered from cold sensitivity (2). To our knowledge, few studies have been performed on the presence of cold sensitivity in nerve compression syndromes. Since QuickDASH, which is often used clinically, does not evaluate cold sensitivity, there is a risk that cold sensitivity symptoms go unrecorded. Further, as the occurrence of cold sensitivity among people with conservative treatment is unknown this could be the subject for future research.

People with diabetes suffered from worse cold sensitivity preoperatively than those without diabetes. There were also significantly more persons with diabetes in the group with severe cold sensitivity than in the groups of mild and moderate cold sensitivity. These findings correspond to the findings of Wendt et al. (2), who concluded that people with diabetes in UNE or CTS report worse cold sensitivity. It is possible that diabetes increases symptoms of cold sensitivity in the presence of a nerve compression lesion. Persons with and without diabetes recovered after surgery to the same extent regarding cold sensitivity symptoms. Surgical treatment for UNE may thus be recommended in persons with diabetes and cold sensitivity.

According to Swedish studies, cold sensitivity affects 5-14% of the normal population, with the highest prevalence in the northern, and generally colder, parts of Sweden (17, 18). It is plausible that the same should apply to people with UNE and cold sensitivity. Since our study included persons from the whole of Sweden, the risk of selection bias is reduced thus strengthening our findings. It would also be interesting to compare our results with a study performed in a warmer country.

Despite our research being based on the largest available register in Sweden concerning UNE, the main limitation of our study is the low response rate. Probably, this is partly due to the difficulty in motivating people to answer questionnaires. As sample size decreases, sub-analyses become more difficult to power properly, and a low response rate elevates the risk of selection bias. Motivation might drive some people to be more likely to participate than others. For instance, those with worse symptoms may be more prone to respond preoperatively, and postoperatively; it is possible that very pleased or dissatisfied people are more interested in answering surveys. Unfortunately, this potential bias is hard to fully evaluate in retrospect. Instead, we compared the characteristics of responders and non-responders. We saw no differences preoperatively, thus the surveys seem to be equally accessible for all ages. However, since scoring on QuickDASH differs between sexes and ages, the increased age and the majority of women among responders postoperatively might skew the results. To circumvent that as far as possible, we adjusted the linear regression analysis for sex and age. We also had no data on preoperative neurography testing; thus, no information about the presence of axonal degeneration. In future studies, it could be valuable to study the correlation between preoperative neurography values and other parameters e.g. function of different types of nerve fibres with various diameters, and cold sensitivity as well as prediction of outcome.

## Conclusion

Cold sensitivity is common in people with UNE and those with diabetes report even more preoperative problems than the rest of the population. The severity of cold sensitivity improves after surgical treatment of UNE in all patients regardless of diabetes. A higher preoperative degree of cold sensitivity is associated with more perceived disability both pre- and post-operatively.

## Data Availability Statement

Public access to data is restricted by the Swedish Authorities (Public Access to Information and Secrecy Act; https://government.se/information-material/2009/09/public-access-to-information-and-secrecy-act/), but data can be available for researchers after a special review that includes approval of the research project by both an Ethics Committee and the authorities’ data safety committees.

## Ethics Statement

The studies involving human participants were reviewed and approved by Regional Ethical Review Board in Lund, Sweden 2016/931, 2018/57 and 2018/72. The patients/participants provided their written informed consent to participate in this study.

## Author Contributions

All authors interpreted the data and critically reviewed the report. MZ and HP collected and analyzed the data as well as drafted the first manuscript. A-MS and KE-O were responsible for collecting the data from the diabetes register. MZ, EN, and LD generated the hypothesis and outline of the project. A-MS and KE-O contributed to hypothesis generation and to writing the manuscript. All authors fulfilled the criteria for authorship. All authors contributed to the article and approved the submitted version.

## Funding

This work was supported by grants from Lund University, the Swedish Diabetes Foundation, Kockska stiftelsen, Skåne University Hospital, and by ALF Grants in Region Skåne and Region Östergötland (register number LIO-823361), Sweden.

## Conflict of Interest

The authors declare that the research was conducted in the absence of any commercial or financial relationships that could be construed as a potential conflict of interest.

## Publisher’s Note

All claims expressed in this article are solely those of the authors and do not necessarily represent those of their affiliated organizations, or those of the publisher, the editors and the reviewers. Any product that may be evaluated in this article, or claim that may be made by its manufacturer, is not guaranteed or endorsed by the publisher.
